# A 6-Gene Risk Signature Predicts Survival of Glioblastoma Multiforme

**DOI:** 10.1155/2019/1649423

**Published:** 2019-08-20

**Authors:** Jingwei Zhao, Le Wang, Guozhang Hu, Bo Wei

**Affiliations:** ^1^Department of Neurosurgery, China-Japan Union Hospital of Jilin University, Changchun, Jilin, 130033, China; ^2^Department of Ophthalmology, The First Hospital of Jilin University, Jilin University, Changchun, Jilin, 130021, China; ^3^Department of Emergency Medicine, China-Japan Union Hospital of Jilin University, Changchun, Jilin, 130033, China

## Abstract

**Background:**

This study aims to develop novel signatures for glioblastoma multiforme (GBM).

**Methods:**

GBM expression profiles from The Cancer Genome Atlas (TCGA) were downloaded and DEGs between tumor and normal samples were identified by differential expression analysis (DEA). A risk signature was developed by applying weighted gene coexpression network analysis (WGCNA) and Cox regression analysis. Patients were divided into high and low risk group, followed by evaluating the performance of the signature via Kaplan-Meier curve analysis. In addition, the prognostic significance of the signature was further validated using an independent validation dataset from Chinese Glioma Genome Atlas (CGGA). DEGs between high and low risk group were subjected to functional annotation.

**Results:**

A total of 748 DEGs were identified between primary tumor and normal samples. Following WGCNA and Cox regression analysis, 6 DEGs were identified and used to construct a risk signature. The signature showed high performance in both training and validation dataset. Subsequently, 397 DEGs were identified between high and low risk group. These DEGs were mainly enriched in terms related to calcium signaling, cAMP-mediated signaling, and synaptic transmission.

**Conclusions:**

The risk signature may contribute to GBM diagnosis in future clinical practice.

## 1. Introduction

As the most malignant and frequently occurring tumor of the central nervous system (CNS), glioblastoma multiforme (GBM) has been considered as a Grade IV glioma according to World Health Organization (WHO) classification [1–3]. Prominent features of GBM include enhanced tumor cell proliferation, migration, and invasion [4]. Recently, the prognosis of GBM has been gradually improved as a result of advances in surgical resection, radiotherapy, and adjuvant chemotherapy [2]. However, GBM remains a deadly tumor with a median survival of only 15 months [2, 5].

Gene expression aberrations are universal events in cancers and may contribute to cancer development and progression [6]. For example, amplification and overexpression of epidermal growth factor receptor (*EGFR*) is found in more than 30% of GBM [7]. It has been shown that GBM tumor cells with EGFR amplification have higher infiltration ability and inhibition of EGFR activity suppresses tumor cell growth [8]. In addition, the expression level of inhibitor of growth family member 4 (*ING4*), which may inhibit tumor cell growth by suppressing nuclear factor kappa B (NF-*κ*B) signaling, has been significantly reduced in GBM [9].

Recent advancements in bioinformatics and high-throughput sequencing have led to the identification of numerous tumor biomarkers, which may allow for more accurate outcome prediction and better management of GBM [10, 11]. Sreekanthreddy et al. identified serum osteopontin (*OPN*) as a biomarker of GBM [12]. High level of* OPN* was confirmed as an indicator of poor outcome of GBM [12]. Colman et al. proposed a 9-gene signature as a predictor of GBM outcome, which showed a close association with markers of glioma stem like cells, including nestin and CD13 [13]. Besides, Arimappamagan et al. established a 14-gene prognostic signature with high accuracy in distinguishing high risk GBM patients from low risk patients [14]. These markers may be integrated into state-of-the-art diagnosis and decision-making processes in future clinical practice. Despite these progressions, more robust prognostic predictors are still needed for GBM treatment.

In our study, we analyzed the expression data of GBM from The Cancer Genome Atlas (TCGA) and Chinese Glioma Genome Atlas (CGGA) and identified differentially expressed genes (DEGs) by differential expression analysis. Subsequently, a 6-gene signature was identified by weighted gene coexpression network analysis (WGCNA) and Cox regression analysis. The signature showed high performance in predicting GBM clinical outcome and may serve as a novel predictor of GBM outcome in future clinical practice.

## 2. Materials and Methods

### 2.1. Data Source

The GBM expression dataset (Illumina HiSeq 2000 RNA Sequencing) from TCGA (https://portal.gdc.cancer.gov/) was downloaded in February, 2019. A total of 173 samples (154 primary tumors, 14 recurrent tumors, and 5 solid tissue normal samples) were included in the dataset. Primary tumor and solid tissue normal samples were selected as training samples for further study. Another GBM expression dataset under accession code “part D” [15] were downloaded from CGGA (http://cgga.org.cn/). The dataset included a total of 272 GBM samples, 138 of which were used as validation samples.

### 2.2. Screening of DEGs

DEGs between primary tumor and solid tissue normal groups were screened using the limma package (version 3.34.7, https://bioconductor.org/packages/release/bioc/html/limma.htm) of R3.4.1 [16]. The selection criteria were fold discovery rate (FDR) < 0.5 and |log_2_⁡FC  (fold  change)| > 0.5. Based on the expression values of the DEGs, two-way hierarchical clustering analysis was performed using pheatmap (version 1.0.8, https://cran.r-project.org/web/packages/pheatmap/index.html) [17].

### 2.3. WGCNA

WGCNA is a bioinformatic method based on high throughput expression data, which is used for the construction of coexpression network [18]. All the expressed genes from TCGA dataset were subjected to WGCNA (version 1.61, https://cran.r-project.org/web/packages/WGCNA/) [18]. Specifically, Pearson coefficients were generated for all pairwise comparisons of genes and the resulting coexpression matrix was transformed to an adjacency matrix using soft threshold power. The soft threshold power was the value where the square of the correlation coefficient between log_2_⁡k (k, the number of connected nodes) and log_2_⁡p(k) (p(k), the probability of k connected nodes) reached 0.9. Subsequently, in order to group genes into different modules, the dissimilarities between genes in the adjacency matrix were generated and hierarchical clustering was performed using the dynamic hybrid tree-cutting method (cutHeight = 0.9; the minimum module size = 50).

The DEGs were then mapped to the WGCNA modules. A hypergeometric algorithm was used to calculate the value of fold enrichment [19], which was defined as

(1) $$\begin{array}{c} f\left(\;k,N,M,n\;\right)=C\left(\;k,M\;\right)*\frac{C\left(n-k,N-M\right)}{C\left(N,M\right)}, \end{array}$$

where *N* indicated the total number of genes analyzed by WGCNA, *M* indicated the number of genes in each module, *n* indicated the number of DEGs and *k* indicated the number of DEGs mapped to each module. The modules with fold enrichment > 1 and p < 0.05 were selected as disease associated modules.

### 2.4. Identification of GBM Associated Gene Signature

In order to identify a GBM related signature genes, univariate and multivariate Cox regression analysis were performed using the survival package (version 2.41-1, https://cran.r-project.org/web/packages/survival/index.html) of R3.4.1. The selection criterion was log-rank p < 0.05. Based on the expression levels of the signature genes, a risk signature was then formulated as

(2) $$\begin{array}{c} risk\;\;score=\sum \beta_{{DEG}_{n}}\times {Exp}_{{DEG}_{n}}, \end{array}$$

where *β*_*DEG*_*n*__ indicated the coefficient of *DEG*_*n*_ derived from multivariate Cox regression whereas *Exp*_*DEG*_*n*__indicated the expression level of *DEG*_*n*_.

The risk score of each sample was calculated according to the above formula. The median of risk score value was used as the threshold to divide the training samples into high and low risk group. The prognostic significance of the risk signature was assessed by Kaplan-Meier curve analysis using the survival package. Subsequently, the prognostic significance of the risk signature was validated using the CGGA dataset with the same procedure.

### 2.5. Functional Characterization of the Different Prognosis

DEGs between high and low risk group of the training dataset were further screened by limma package. The selection criteria for DEGs were FDR < 0.5 and |log_2_⁡FC| > 0.5. The resulting DEGs were subjected to functional annotation analysis using the clusterProfiler package (http://bioconductor.org/packages/release/bioc/html/clusterProfiler.html) of R3.4.1 [20]. The selection criterion for GO biological processes and KEGG pathways was FDR < 0.05.

## 3. Results

### 3.1. DEGs between Tumor and Normal Samples

**Figure 1:**
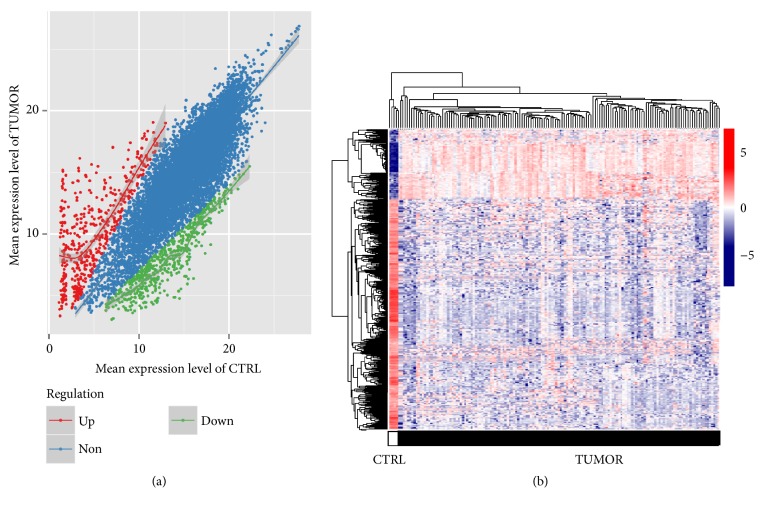
*Differentially expressed genes (DEGs) and bidirectional hierarchical clustering*. (a) Scatter plot showing gene expression levels. Upregulated and downregulated DEGs are shown as red and green dots, respectively. Genes with no obvious changes in expression level are shown as blue dots. (b) Bidirectional hierarchical clustering of samples based on the expression level of DEGs. Tumor and normal samples are represented as dark and white bar, respectively. Upregulated and downregulated DEGs are shown in red and blue, respectively.

Following differential expression analysis, a total of 748 DEGs (218 upregulated and 530 downregulated) (Figure 1(a)) were identified between primary tumor and solid tissue normal samples. Then the specificity of the DEGs was evaluated by two-way hierarchical clustering analysis. According our results, primary tumor and solid tissue normal samples were divided into two clusters, which showed completely different overall expression patterns (Figure 1(b)).

### 3.2. Disease Related WGCNA Modules and Genes

**Figure 2:**
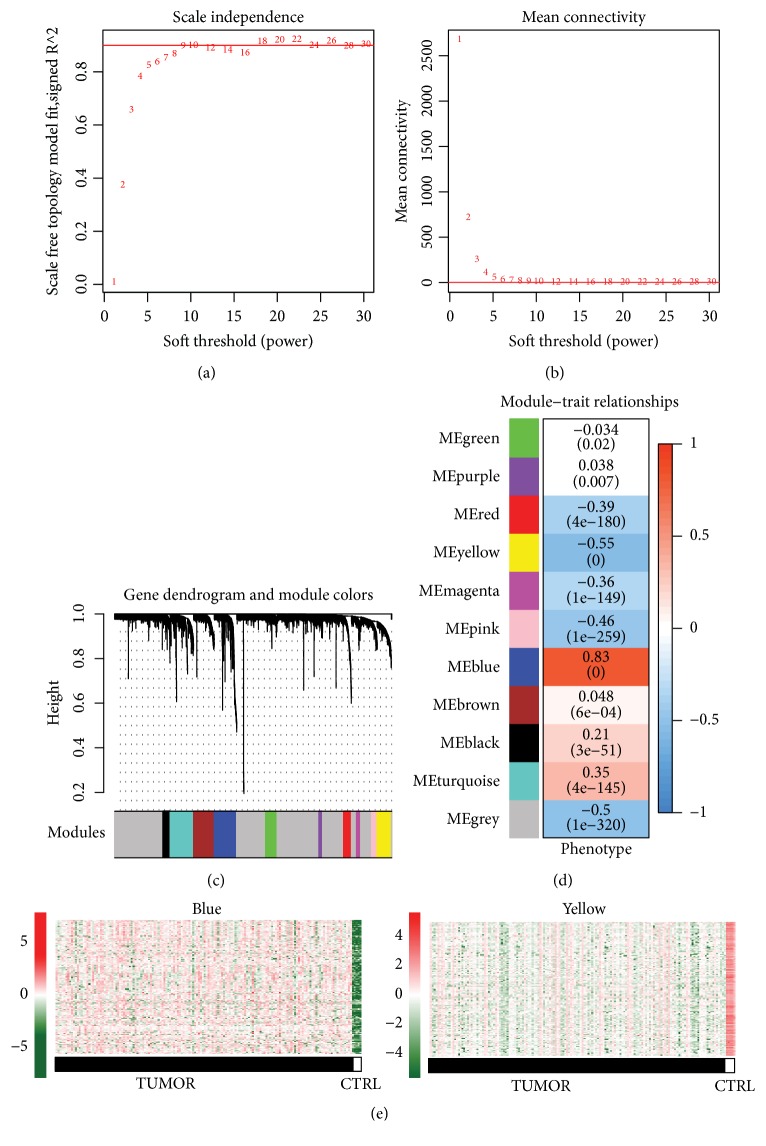
*Coexpression modules revealed by weighed gene co-expression network analysis (WGCNA)*. (a) Determination of soft threshold for adjacency matrix. The horizontal axis represents the soft threshold power and the vertical axis represents the square of the correlation coefficient of between log_2_⁡k and log_2_⁡p(k). The red line indicates where the correlation coefficient is 0.9, and the corresponding soft threshold power is 18. (b) Plots of mean connectivity versus soft threshold. The red line indicates where power is 18, and the corresponding mean connectivity is 1.0. (c) Gene dendrogram derived from hierarchical clustering. Different modules are indicated by colors underneath the dendrogram. (d) Module-trait relationships. Along the vertical axis are the modules represented by different colors. Correlation coefficients are shown as numbers in corresponding positions and p values are shown in brackets along the coefficients. (e) Heatmap of DEGs in blue (left) and yellow (right) modules. Tumor and normal samples are represented as dark and white bar, respectively. Upregulated and downregulated DEGs are shown in red and green, respectively.

All the expressed genes from TCGA dataset were used as input for WGCNA. The soft threshold power used for matrix transformation was determined as 18, where the square of the correlation coefficient between log_2_⁡k and log_2_⁡p(k) reached 0.9 and the mean connectivity of the co-expression network was 1.0 (Figures 2(a) and 2(b)). According to the WGCNA results, totally 10 different disease related modules were obtained, except for the grey module (Figure 2(c)). The correlations between WGCNA modules and disease were shown as heatmap in Figure 2(d).

A total of 745 overlap genes were obtained after mapping DEGs to WGCNA modules (Table 1). According to hypergeometric algorithm analysis, DEGs were significantly enriched in blue and yellow module (fold enrichment > 1 and p < 0.05), each containing 150 and 136 DEGs, respectively (Table 1). The expression level of these DEGs in primary tumor and solid tissue normal samples were shown as heatmaps in Figure 2(e).

### 3.3. GBM Associated Risk Signature

In total, 152 of the primary tumor samples had clinical prognosis information and were used for subsequent screening of signature genes from DEGs in the blue and yellow module. Univariate and multivariate Cox regression analysis were performed sequentially. A total of 27 DEGs were obtained by univariate Cox regression, among which 6 DEGs were further identified as prognosis associated genes by multivariate Cox regression (Table 2). The 6 DEGs were BPI fold containing family B member 2 (*BPIFB2*), Xin actin binding repeat containing 2 (*XIRP2*), leucine rich repeat containing 10 (*LRRC10*), short chain dehydrogenase/reductase family 16C member 5 (*SDR16C5*), Homeobox A13 (*HOXA13*), and neural EGFL like 1 (*NELL1*).

The 6 DEGs were signature genes and were used to develop a tumor associated risk signature. According to the *β* values (Table 2) and the expression levels of these genes, risk score could be defined as

(3) risk  score=−0.0881∗ExpBPIFB2−0.1441∗ExpXIRP2−0.0614∗ExpLRRC10−0.0945∗ExpSDR16C5−0.0854∗ExpHOXA13−0.151∗ExpNELL1.

**Figure 3:**
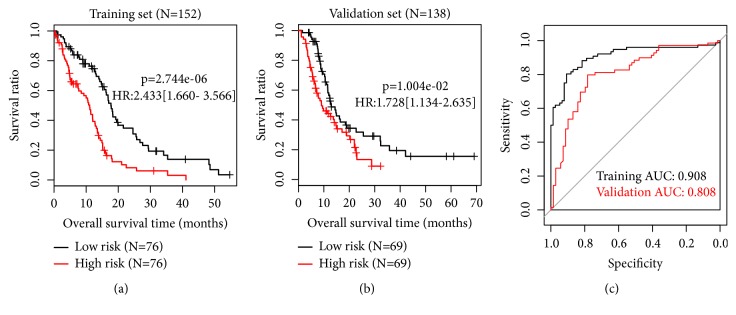
*Survival analysis based on the risk signature*. (a) Kaplan-Meier curves of low (black) and high (red) risk groups in the training dataset. (b) Kaplan-Meier curves of low (black) and high (red) risk groups in the validation dataset. (c) Receiver operating characteristic (ROC) curves for training (black) and validation (red) dataset. Area under ROC curve (AUC) is calculated to be 0.908 and 0.808 for training and validation dataset, respectively.

Risk score of each sample was calculated based on the above formulation. The median risk score was used as the cutoff to separate samples in TCGA dataset into high and low risk group. According to the Kaplan-Meier survival curve, the prognosis of low risk group was significantly better than that of high risk group (Figure 3(a); p < 0.05). The performance of the signature in predicting prognosis was further validated in the validation dataset using the same procedure. Consistent with the result of training dataset, low risk group also showed a significantly better prognosis than high risk group in the validation dataset (Figure 3(b)). Moreover, the validation dataset also showed a high area under the receiver operating characteristic curve (AUC), close to that of the training dataset (Figure 3(c)).

### 3.4. Functional Annotation of Feature Genes

**Figure 4:**
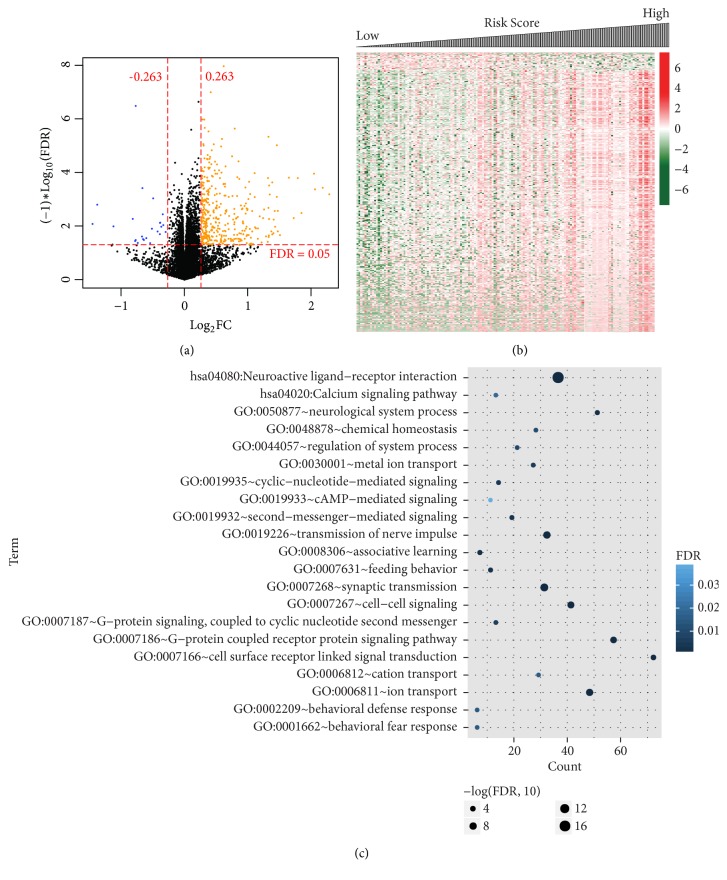
*Functional annotation of signature genes*. (a) Volcano plot of expressed genes. Orange and blue dots indicate upregulated and downregulated DEGs, respectively. Black dots indicate genes with no significant changes in expression level. The horizontal red dash line indicates where false discovery rate (FDR) = 0.05 and the vertical red dash lines indicate where |log_2_⁡FC  (fold  change)| = 0.263. (b) Heatmap showing the expression levels of DEGs. Downregulated and unregulated genes are shown in green and red, respectively. (c) Gene Ontology (GO) and Kyoto Encyclopedia of Genes and Genomes (KEGG) terms enriched by DEGs between high and low risk group. Pathway and functional terms (vertical axis) are plotted against genes numbers (horizontal axis). The values of –lg(FDR) are in proportion to the sizes of dots.

A total of 397 DEGs (371 upregulated and 26 downregulated) between low and high risk group of the training dataset were screened by differential expression analysis (Figure 4(a)). The expression patterns of these DEGs were shown as heatmap in Figure 4(b).

In order to interpret the biological functions and pathways perturbated by the signature genes, the DEGs between low and high risk group were used as inputs for GO and KEGG analysis. The enriched KEGG pathways were “hsa04080: Neuroactive ligand-receptor interaction” and “hsa04020: Calcium signaling pathway” (Figure 4(c)). The main enriched GO biological processes included “GO:0019933~cAMP−mediated signaling” “GO:0006811~ion transport” “GO:0007268~synaptic transmission” and “GO:0019226~transmission of nerve impulse” (Figure 4(c)).

## 4. Discussion

GBM is the most malignant brain tumor, efficient management of which requires robust biomarkers [11]. In the present study, we first analyzed the expression dataset of GBM and identified DEGs between primary tumor and solid tissue normal samples. Based on the DEGs, we successfully developed a risk signature, which was efficient and reliable in predicting the clinical outcome of GBM.

Signatures composed of multiple genes are generally more robust and more accurate than single-gene biomarker in predicting tumor outcome [14]. The risk signature developed in our study consisted of 6 genes, including* BPIFB2*,* XIRP2, LRRC10, SDR16C5*,* HOXA13,* and* NELL1*. GBM samples could be divided into high and low risk group by applying the signature. As expect, our results indicated that low risk groups showed significantly better overall survival than high risk groups in both training and validation dataset. Consequently, the 6-gene signature may provide useful information for clinical practice if incorporated into future decision-making processes.

Among the signature genes,* HOXA13* is a Homeobox gene overexpressed in multiple cancers and has been shown to be associated with the progression of hepatocellular carcinoma, pancreatic cancer, esophageal squamous cell carcinoma and GBM [21]. It has been reported that* HOXA13* may promote GBM progression through activation of Wnt/beta-catenin and TGF-*β* signaling pathway, whereas downregulation of* HOXA13* may suppress GBM cell invasion and decrease tumor growth [22]. Though few studies have investigated roles of* BPIFB2*,* XIRP2* and* NELL1 *in GBM, all the three genes may have potential roles in other cancers.* BPIFB2* has been reported to be overexpressed in gastric cancer, which may result in expression alteration of epithelial-mesenchymal transition (EMT) markers [23], and* XIRP2 *has been shown to be frequently mutated in lung adenocarcinoma [24]. As a gene encoding a secreted protein regulating skeletal ossification [25],* NELL1* has also been proposed to be a tumor suppressor gene in colon cancers [26]. Therefore, dysregulation of the* BPIFB2*,* XIRP2* and* NELL1* may also underly GBM tumorigenesis. While the roles of the remaining two signature genes in GBM tumorigenesis remains unclear, both genes have important physiological roles.* LRRC10 *is required for early heart development [27, 28] and* SDR16C5* encodes a retinol dehydrogenase, which may be essential for retinoic acid biosynthesis [29]. Considering their important physiological roles, they may also potentially be involved in GBM tumorigenesis.

Dysregulation of cancer related pathways and functions is common in cancers [30]. Our functional annotation analysis showed that DEGs between high and low risk group were significantly enriched in pathways and functions related to synaptic transmission, indicating that synaptic function in GBM patients may be deregulated. In addition, DEGs were also enriched in calcium signaling and cAMP-mediated signaling. Ca^2+^ is an essential regulator for neurogenesis and synaptic transmission, and the deregulation of Ca^2+^ signaling may advance GBM progression [31, 32]. It has been proposed that manipulating Ca^2+^ signaling may benefit the management of GBM [31]. Suppression of cAMP signaling pathway has been shown to be a common feature in tumorigenesis and activation of cAMP signaling in GBM may inhibit tumor cell growth and induce cell apoptosis [33]. Considering the prognostic differences between high and low risk group, we speculated that deregulation of calcium signaling and cAMP-mediated signaling may play important roles in the development and progression of GBM.

One main advantage of our study was that a robust GBM risk signature was developed through a combination of WGCNA and Cox regression analysis. The signature was efficient and reliable for both training and validation dataset when applied in outcome prediction. In addition, we also identified* BPIFB2*,* XIRP2*,* NELL1*,* LRRC10, *and* SDR16C5* as novel GBM biomarkers, as they have never been reported to be associated with GBM development and progression. However, we also noticed some limitations in our study. The samples included in our study was insufficient and more samples are required in future studies. Additionally, experimental studies should be designed and performed to confirm the involvement of the novel biomarkers in GBM and to provide an insight into corresponding molecular mechanisms.

## 5. Conclusion

In conclusion, we analyzed the expression profiles of GBM and identified DEGs between primary tumor and solid tissue normal samples. A 6-gene risk signature consisting of* BPIFB2*,* XIRP2, LRRC10, SDR16C5*,* HOXA13,* and* NELL1* was further developed for outcome prediction. The signature may contribute to future decision-making processes in clinical practice.

## Figures and Tables

**Table 1:** Statistical data of WGCNA modules.

Module	Total gene number	DEG number	Enrichment fold (95% CI ^a^ )	P_hyper_
black	132	4	0.203(0.054-0.535)	1.21E-04
blue	405	150	2.484(2.015-3.053)	2.20E-16
brown	374	12	0.215(0.109-0.383)	7.61E-11
green	211	0	-	-
grey	2814	380	0.906(0.792-1.035)	1.52E-01
magenta	78	6	0.516(0.183-1.181)	1.38E-01
pink	101	12	0.797(0.397-1.463)	5.71E-01
purple	67	0	-	-
red	140	13	0.623(0.322-1.108)	1.12E-01
turquoise	422	32	0.508(0.341~0.736)	1.20E-04
yellow	255	136	3.578(2.843-4.489)	2.20E-16

^a^ Confidence interval.

**Table 2:** DEGs identified by multivariate Cox regression.

ID	*β*_DEG_	HR ^a^	95% CI ^b^	p
BPIFB2	-0.0881	0.9157	0.8598-0.9753	0.00619
XIRP2	-0.1441	0.8658	0.7746-0.9677	0.01112
LRRC10	-0.0614	0.9404	0.8926-0.9908	0.02095
SDR16C5	0.0945	1.0991	1.0077-1.1989	0.03299
HOXA13	-0.0854	0.9182	0.8468-0.9955	0.03847
NELL1	0.1510	1.1630	1.0032-1.3482	0.04526

^a^ Hazard ratio.

^b^ Confidence interval.

## Data Availability

No data were used to support this study.
